# Mechanisms of Immune Dysregulation in COVID-19 Are Different From SARS and MERS: A Perspective in Context of Kawasaki Disease and MIS-C

**DOI:** 10.3389/fped.2022.790273

**Published:** 2022-05-05

**Authors:** Manpreet Dhaliwal, Rahul Tyagi, Pooja Malhotra, Prabal Barman, Sathish Kumar Loganathan, Jyoti Sharma, Kaushal Sharma, Sanjib Mondal, Amit Rawat, Surjit Singh

**Affiliations:** Allergy and Immunology Unit, Department of Pediatrics, Advanced Pediatrics Center, Postgraduate Institute of Medical Education and Research, Chandigarh, India

**Keywords:** SARS-CoV, MERS-CoV, SARS-CoV-2, COVID-19, Kawasaki disease, MIS-C, immune dysregulation

## Abstract

Coronaviruses have led to three major outbreaks to date-Severe Acute Respiratory Syndrome (SARS; 2002), Middle East Respiratory Syndrome (MERS; 2012) and the ongoing pandemic, Coronavirus Disease (COVID-19; 2019). Coronavirus infections are usually mild in children. However, a few children with MERS had presented with a severe phenotype in the acute phase resulting in progressive pneumonic changes with increasing oxygen dependency and acute respiratory distress requiring ventilatory support. A subset of children with a history of SARS-CoV-2 infection develops a multisystem hyper-inflammatory phenotype known as Multisystem Inflammatory Syndrome in Children (MIS-C). This syndrome occurs 4-6 weeks after infection with SARS-CoV-2 and has been reported more often from areas with high community transmission. Children with MIS-C present with high fever and often have involvement of cardiovascular, gastrointestinal and hematologic systems leading to multiorgan failure. This is accompanied by elevation of pro-inflammatory cytokines such as IL-6 and IL-10. MIS-C has several similarities with Kawasaki disease (KD) considering children with both conditions present with fever, rash, conjunctival injection, mucosal symptoms and swelling of hands and feet. For reasons that are still not clear, both KD and MIS-C were not reported during the SARS-CoV and MERS-CoV outbreaks. As SARS-CoV-2 differs from SARS-CoV by 19.5% and MERS by 50% in terms of sequence identity, differences in genomic and proteomic profiles may explain the varied disease immunopathology and host responses. Left untreated, MIS-C may lead to severe abdominal pain, ventricular dysfunction and shock. Immunological investigations reveal reduced numbers of follicular B cells, increased numbers of terminally differentiated CD4^+^T lymphocytes, and decreased IL-17A. There is still ambiguity about the clinical and immunologic risk factors that predispose some children to development of MIS-C while sparing others. Host-pathogen interactions in SARS, MERS and COVID-19 are likely to play a crucial role in the clinical phenotypes that manifest. This narrative review focuses on the immunological basis for development of MIS-C syndrome in the ongoing SARS-CoV-2 pandemic. To the best of our knowledge, these aspects have not been reviewed before.

## Introduction

Over the last two decades, coronaviruses have become a significant threat to humans. The previous two outbreaks caused by the β-coronaviruses genera, viz. Severe Acute Respiratory Syndrome (SARS) in 2002, and Middle East Respiratory Syndrome (MERS) in 2012, were confined to China and Middle East and East Asia, respectively. The current outbreak, viz. Coronavirus Disease (COVID-19) is caused by SARS-CoV-2, which originated in Wuhan, China and soon turned into a pandemic. It has brought into focus the significant health threat posed by these viruses. As of now, more than 264 million cases of COVID-19 have been reported encompassing virtually every known country. With over 5.2 million deaths, this pandemic has caused severe strain to the existing healthcare system, especially in developing countries (161).

In late April 2020, children in Europe, and later in North America, were reported to develop high grade fever, rash, conjunctival injection, gastrointestinal manifestations, myocarditis, features of hyperinflammation, and in some cases coronary artery aneurysms (CAAs) 4–6 weeks after SARS-CoV-2 infection. There was uncertainty whether these symptoms were related to atypical Kawasaki Disease (KD), Kawasaki disease shock syndrome (KDSS) (1) or toxic shock syndrome (TSS). This unique cluster of symptoms was designated as “Paediatric Multisystem Inflammatory Syndrome temporally associated with SARS-CoV-2 (PIMS-TS),” in the United Kingdom, or “Multisystem Inflammatory Syndrome in Children (MIS-C) associated with COVID-19” by the Centers for Disease Control and Prevention (CDC), Atlanta, United States.

In a large cohort study from the United States, the cumulative incidence of MIS-C was found to be 2.1 per 100,000 individuals younger than 21 (2, 3). The incidence varied from 0.2 to 6.3 per 100,000 across different states. A mortality rate of 1.4 percent was recorded (3). In May 2020, the World Health Organization (WHO) defined MIS-C based on pilot case reports to assist diagnosis of this disease as a post-COVID complication. Though the clinical similarities and differences of MIS-C with KD have been discussed in literature, the molecular basis of immune dysregulation in MIS-C in comparison to previous CoV related pandemics has not been discussed in detail.

Host-pathogen interactions in SARS, MERS and COVID-19 are likely to play a crucial role in the clinical phenotypes that manifests as KD or MIS-C. These characteristics were unique since sequela like MIS-C were not witnessed in earlier CoV outbreaks (SARS and MERS). We aim to review immunological mechanisms governing the display of KD like MIS-C phenotype. This review provides new insights into the complex interplay between these infective and inflammatory disorders.

## Taxonomy of Coronaviruses

Coronaviruses (CoVs) have historically been implicated in 15–30% of all common colds, but have now also been associated with other severe illnesses such as croup, exacerbations of asthma and bronchiolitis (4–6). Presently, 7 strains of coronaviruses have been found to be pathogenic for humans (7).

**FIGURE 1:**
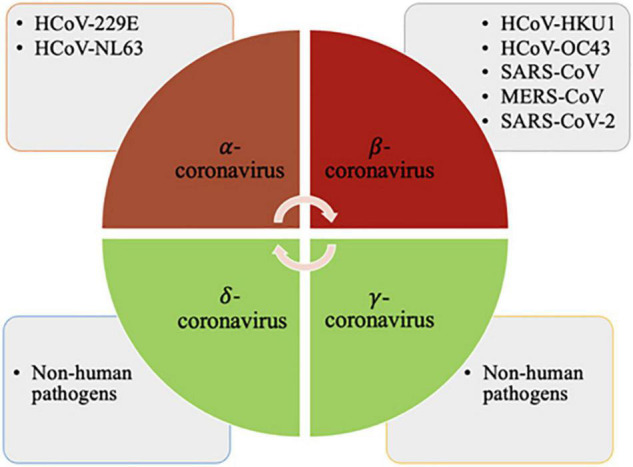
The α-coronaviruses are HCoV-229E, HCoV-NL63; β-coronaviruses are HCoV-HKU1, HCoV-OC43, SARS-CoV, MERS-CoV and SARS-CoV-2; and the γ and δ-coronaviruses comprise species that are non-pathogenic to humans. Created with BioRender.com.

The coronaviruses are enveloped viruses that are highly variable in size (80–220 nm). The genetic material consists of single-stranded positive-sense RNA. CoVs belong to the order-Nidovirales, family-Coronaviridae, and subfamily- Orthocoronavirinae. These are enveloped single stranded positive-sense RNA viruses capable of infecting both vertebrates and invertebrates and are divided into four major genera i.e., α, β, γ and δ-coronaviruses (Figure 1).

## Clinical Characteristics and Epidemiology of COVID-19, Kawasaki Disease and Multisystem Inflammatory Syndrome in Children

**FIGURE 2:**
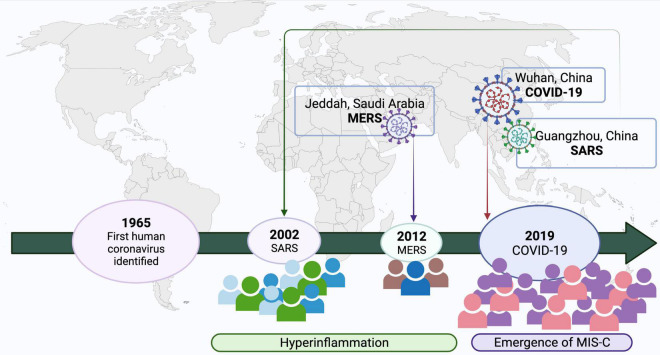
Emergence of coronavirus in humans and relationship with KD-MISC spectrum. Created with BioRender.com.

The α-coronavirus and most β-coronaviruses are implicated in mild respiratory illnesses. However, a group of β-coronaviruses which includes SARS-CoV, MERS-CoV and SARS-CoV-2 have caused major outbreaks and pandemics since the beginning of the 21st century. SARS-CoV-2 has resulted in the ongoing pandemic that has affected all age groups. Comparative details of the three pandemics caused by CoVs have been provided in Table 1 and Figure 2. A new entity, MIS-C was described by various reports at the onset of COVID-19 pandemic post SARS-CoV-2 infection (8, 9).

**TABLE 1:** Clinical characteristics and epidemiology of SARS, MERS and COVID-19.

Details	SARS	MERS	COVID-19
Year first reported; Country	November, 2002; Foshan, Guangzhou, China	June, 2012; Jeddah, Saudi Arabia	December 2019; Wuhan, China
Total number of cases	8,096 with 774 deaths	2,574 with 886 deaths	Ongoing, ∼ 234 million infected with 4.8 million deaths
Total number of pediatric cases	135	42	∼ 8.5% of cases are children
Total pediatric deaths	None	3	Globally 3,788 deaths till January, 2021 (138); 561 deaths in United States till September, 2021 (CDC, United States)
Mortality rate	9.5%	34.4%	∼ 2.5%
Putative reservoir of infection	Asian civet cat (*Paguma larvata*)	Adult dromedary camels (*Camelus dromedarius*)	?Bat; pangolins; snakes
Mode of Transmission	Human to human—High; direct contact and Indirect contact (droplets or fomites, aerosol transmission and rarely faeco-oral transmission)	Human to human—Low; direct contact with infected camels; close contact with patients	Human to human—High; droplet infection; fomites; aerosols; close contact with patients
Putative Host receptor for viral entry	ACE2	DPP4	ACE2
Treatment	Mainly supportive treatment; ribavirin, steroids were used anecdotally	Mainly supportive treatment	Mainly supportive treatment Following drugs have been tried with variable efficacy: remdesivir, corticosteroids, hydroxychloroquine, lopinavir/ritonavir, ivermectin, tocilizumab, anakinra
Incubation period in days	2–10 (mean: 6.4)	2–13 (mean: 5)	2–14 (mean: 5)
Hematological and biochemical findings	Lymphopenia; thrombocytopenia; hypoalbuminemia; transaminitis; increased lactic dehydrogenase, creatine kinase and C-reactive protein levels	Similar to SARS	Similar to SARS along with elevated D-Dimer, and IL-6

*ARDS, acute respiratory syndrome; MODS, multi-organ dysfunction syndrome; ACE-2, angiotensin-converting enzyme-2; DPP4, dipeptidyl-peptidase-4.*

Kawasaki disease and MIS-C phenotypes overlap in terms of few clinical presentations such as fever, rash, erythema and edema. Previously, a case-control study on Kawasaki disease demonstrated the presence of New Haven coronavirus (HCoV-NH) by a reverse-transcriptase polymerase chain reaction in respiratory secretions of 72.7% KD patients (10). This was taken to be proof of KD being a sequela of viral infection. However, broader epidemiological confirmation, in-depth evaluation of immune response to HCoV-NH, and presence of the virus in biopsy is required to confirm this agent as the causative agent of KD (11). In the current pandemic, KD like symptoms was observed in the pediatric cases of COVID-19, later named as a MIS-C (12). MIS-C diverge to be a distinct pathophysiological mechanism with severe multisystemic hyperinflammation especially myocarditis with cardiac dysfunction. Laboratory parameters suggestive for the diagnosis of MIS-C include serum ferritin, leukopenia, lymphopenia (13). Prognosis in MIS-C is calculated by taking into account cardiovascular complications, such as presence of ventricular dysfunction and coronary artery aneurysms (CAA). CAA occurs in 9–24% of cases with MIS-C (14, 15). These complications have triggered recommendations for immunomodulatory treatments, including intravenous immunoglobulin (IVIG), corticosteroids, biologics, and recommendations for intensive cardiac observation. The choice of IVIG treatment was mainly considered based on similarity to Kawasaki disease (15).

Interestingly, presentation similar to MIS-C was not reported in SARS or MERS. Limited transmission of SARS-CoV and MERS-CoV in previous outbreaks, which were largely endemic, may be a possible explanation to non-emergence of a MIS-C like illness. Another possibility could be that the antigenic and infections determinants of SARS-CoV-2 may be responsible for the MIS-C phenotype. Comparison of demographics, clinical features and laboratory investigations between KD and MIS-C has been summarized in Table 2.

**TABLE 2:** Comparison of demographic and clinical features of Kawasaki disease and SARS-CoV-2 related MIS-C.

Parameters	Kawasaki disease	SARS-CoV-2 related MIS-C
First reported	1967; Tokyo, Japan	April 2020; London, United Kingdom
Trigger	Unknown etiology (Probable infectious trigger in genetically predisposed patients)	SARS-CoV-2
Age group	More common in children < 5 years	4–13 years
Ethnicity	Worldwide; highest incidence in East Asia (Japan, Korea, Taiwan)	Worldwide; paucity of cases in East Asia
Interval between exposure and symptoms	Not known	3–6 weeks
Sex	Male > female	No clear sex bias
Systems involved	Cardiac: Coronary artery aneurysms during convalescent phase (after 3 weeks of fever); myocarditis	Cardiac: Coronary artery aneurysms during acute phase, myocarditis and left ventricular dysfunction CAAs are usually transient
	CNS: Irritability and aseptic meningitis	CNS: Meningitis and encephalitis
	Gastro-intestinal: Gall bladder hydrops	Gastro-intestinal: Abdominal pain (acute pseudo surgical abdomen, peritoneal effusion)
Laboratory parameters	Neutrophilic leukocytosis; thrombocytosis elevated inflammatory parameters (CRP, Procalcitonin, Troponin, Pro-BNP)	Lymphopenia; thrombocytopenia significantly elevated inflammatory parameters (CRP, Procalcitonin, Troponin, Pro-BNP, Ferritin)
Complications	KD shock syndrome (2–7%) MAS (1.3%) (139)	MODS (More than 70%) MAS
Mortality	<0.1% in Japanese cohort	1–2%
Immunological features	High levels of TNF-α, IL-17 Autoantibodies against DEL-1 (anti-inflammatory protein against ICAM-1) Role of IgA in pathogenesis	High levels of plasma—IL-17a Autoantibodies against MAP2K2 and casein kinase family Autoreactive IgG
Treatment	IVIg, steroids, infliximab	IVIg and steroids

*CNS, central nervous system; CRP, C-reactive protein; MODS, multiple organ dysfunction; BNP, brain natriuretic peptide; IVIg, intravenous immunoglobulin; MAS, macrophage activation syndrome.*

## SARS-CoV/MERS-CoV/SARS-CoV-2 Genome and Antigenic Components

**FIGURE 3:**
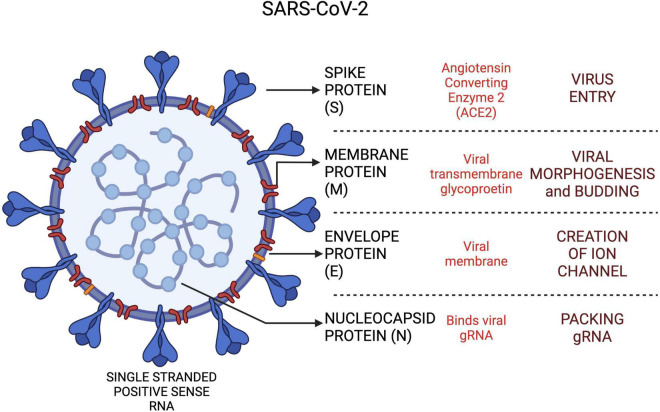
The SARS-CoV-2 viral proteins and functional roles in host cells. Created with BioRender.com.

The CoVs share similar genetic architecture for encoding the proteins involved in the virion structure and transmissions. However, the number and functions of open reading frames (ORFs) differ amongst the CoVs. The comparative structure of SARS-CoV, MERS-CoV and SARS-CoV-2 genome is shown in Figure 3. Order of genes (5′–3′), identified by genomic analysis was as follows: replicase ORF1ab, spike (S), envelope (E), membrane (M) and nucleocapsid (N).

The ORF1ab region spans approximately 67% of the viral genome and contributes to encoding of non-structural proteins (nsps) (16). Nsps have a critical role in CoV RNA synthesis and processing (17). SARS-CoV-2 shares a highly conserved domain of 122–130 amino acid residues with SARS-CoV in nsp1. The spike glycoprotein encoded by the S gene, is recognized as a critical antigenic determinant of host range and pathogenicity as it modulates receptor recognition and cellular entry (18). Cell entry in SARS-CoV-2 is mediated by a carboxypeptidase, angiotensin-converting enzyme 2 (ACE-2), *via* viral spike protein. The S protein is then proteolytically cleaved by a proprotein convertase, furin, into two subunits, S1 and S2, followed by priming of S2 fragment by a host serine peptidase, transmembrane protease serine (TMPRSS2) (19, 20). This novel furin mediated cleavage of the S protein is seen only in SARS-CoV-2 but not in SARS-CoV and MERS-CoV (20). These peptidases serve to unmask a new C-terminal sequence, Arg-Arg-Ala-Arg which facilitates binding of virus to host cells *via* NRP1 receptor (21).

The host secondary receptors in SARS-CoV-2, furin and NRP1 are distinct from SARS-CoV which recruits DC-SIGN and L-SIGN (22). Widespread co-expression of ACE-2 and TMPRSS2 receptors are noted in nasal passages but furin along with ACE-2 and TMPRSS2 are expressed in lung (20). TMPRSS2 belongs to a sub-family of membrane-associated serine protease which along with ACE-2 are expressed by many organ systems. This may explain the enhanced infectivity and exacerbated host response seen in SARS-CoV-2 infection. The spike glycoprotein remains the key target of neutralizing antibodies in the host (23). This protein is also thought to act as a superantigen, causing MIS-C and cytokine storms in adults (24). However, the superantigen property might be related to the configuration of spike protein as SARS-CoV-2 variants evoke variable host immune responses. This phenomenon was observed in the recent omicron variant which was highly infectious but generated a reduced immune response as compared to the delta variant. The SARS-CoV-2 viral proteins and their roles in the host are depicted in Figure 3.

Equilibrium dissociation constant of SARS-CoV-2 has been found to be lower than that of SARS-CoV, indicating substantially different affinity for ACE2 between both CoVs (25). Globally, SARS-CoV-2 has evolved at the rate of two mutations per month (26, 27).

Newly discovered variants of the novel SARS-CoV-2 are thought to be potential triggers for MIS-C as there was a dramatic increase in viral infectivity and pathogenicity following the start of the pandemic. The earliest emerging variants include D614G and N439K (B.1.258). The D614G polymorphism has been associated with the MIS-C phenotype (28). Some polymorphic variants (e.g., D839Y/N/E and A831V) have been predicted to enhance the binding affinity with T cell receptor (TCR). These variants were identified in Europe and North America, and have also been associated with the emergence of MIS-C. A causal relationship between these variants and MIS-C has, however, not been established (29).

The newly emerged omicron (B.1.1.529) variant harbors more than 30 mutations in S- protein alone. Modeling studies revealed that molecular interactions in omicron are more stable than previous variants resulting in enhanced potency of ACE2-spike protein interactions (30). Moreover, the majority of neutralizing mAbs against the omicron variant loses inhibitory activity (31). This variant has unprecedented infectivity, however, pediatric hospitalizations are reduced by half in the omicron wave, unlike the delta variant. The moderate immune response generated in response to the omicron variant may be due to the unmasking of poorly immunogenic spike peptides.

## Host Genetics in Severe COVID-19, Kawasaki Disease and Multisystem Inflammatory Syndrome in Children

Initial SARS-Cov-2 entry is mediated by transmembrane protease serine 2 (TMPRSS2) with one variant (p.Val160Met) reported to be associated with higher viral load and mortality (32). Other components of host immune system, such as, sensing and signaling pathways have also been associated with enhanced infectivity and poor outcomes in SARS-CoV-2 infections. TLR3, Interferon regulatory factor-7 (IRF7), and IRF9 drive type I interferon antiviral responses. These genes are also linked to inborn errors of immunity and individuals with mutations in these genes are predisposed to severe influenza pneumonia (33). Whole exome/genome sequencing based study in 659 cases with severe COVID-19 identified variants in 13 loci, predominantly affecting TLR3 and IRF7 dependent activation of type I IFNs. Zhang et al. proved the association of autosomal recessive *IRF7* deficiency and a failure to mount IFN-I and III responses against SARS-CoV-2 using patient derived plasmacytoid dendritic cells (34). These plasmacytoid dendritic cells mount robust type I interferon response against viral attack. In contrast, amongst the three loci (TLR3, *IRF7* and *IRF9*), Povysil et al. reported association of only one predicted loss of function variant in severe COVID-19 (35). These conflicting reports highlight the need for genome wide association studies (GWAS) with stringent and uniform inclusion/exclusion criteria for better interpretation of genomic data.

Double stranded RNA acts as a ligand for TLR3 which leads to activation of downstream NFκB and IRF3 (36). A population based GWAS reported polymorphism in *TLR3* gene (rs3775291) was associated with increased susceptibility and death in COVID-19 patients (37). Another group of investigators reported that SARS-CoV-2 induces senescence in human cells and amplifies the senescence-associated secretory phenotype (SASP) *via* TLR-3 signaling. These SASP cells produce pro-inflammatory cytokines and damage surrounding tissues (38). Contrary to the observations in human studies, a murine model of lethal influenza infection reported that TLR3-/- mice had longer survival compared to wild type mice. Also, reduced levels of chemokines, fewer infiltrating leukocytes, and less numbers of CD8^+^ T cells were observed in TLR3-/- mice (39). This study cemented the role of TLR3 in ISG induced restriction of viral replication and lung damage due to TLR3 mediated recruitment of immune cells. Functional studies in human patients with polymorphisms in TLR3 are needed to conclusively define the role of TLR3 in COVID-19.

Similar studies in MIS-C discovered few variants in immune related genes, but these variants differed from those reported previously in severe COVID-19. Lee et al. reported presence of heterozygous variant in two unrelated patients in the suppressor of cytokine signaling 1 (*SOCS1*) gene in children presenting with MIS-C (40). SOCS1 is a critical negative regulator of Type I and II IFN signaling. Chou et al. carried out whole exome sequencing to determine genetic risk factors in 18 patients with MIS-C. Genetic defects in X-linked inhibitor of apoptosis (*XIAP*), cytochrome b beta (*CYBB*), and *SOCS1* were detected. However, in 17% of cases, variants affecting the negative regulation of interferon were detected (41). This suggests that there may be a genetic predisposition to development of MIS-C but large scale GWAS remain to be carried out. Though MIS-C and KD share similar clinical features, the genetic risk factors vary amongst these conditions. GWAS studies have linked a number of genetic loci to the pathogenesis of KD, as described in the Table 3.

**TABLE 3:** Genetic loci associated with KD, MIS-C and severe COVID-19.

S. No	Study group	Disease	Associated host gene alterations	Function	Results	References
1	(103)	MIS-C	*SOCS1*	Negative regulator of IFN signaling	2/2 (100%)	(40)
2	(41)	MIS-C	*XIAP, CYBB, and SOCS1*	XIAP regulates cell death, and CYBB is essential in phagocytic NADPH-oxidase activity	5/18 (27.77%)	(41)
3	(140)	KD	*FCGR2A, ITPKC and rs2233152*	*FCGR2A*: Encodes IgG immunoglobulin Fc receptor ITPKC: Regulates calcium channels and controls the activated state of T cells	*FCGR2A: P* = 7.35 × 10 (−11), odds ratio (OR) = 1.32 *rs2233152: P* = 2.51 × 10 (−9), OR = 1.42 *ITPKC: P* = 1.68 × 10 (−12), OR = 1.52	(140)
4	(141)	KD	*FAM167A-BLK* (rs2254546) *HLA* (rs2857151), *CD40* (rs4813003), and *FCGR2A* (rs1801274)	BLK:B cell proliferation and differentiation HLA: Regulation of immune system CD40: co-stimulatory protein found on antigen-presenting cells	rs2254546: *P* = 8.2 × 10 (−21) rs2857151: *P* = 4.6 × 10 (−11) rs4813003: *P* = 4.8 × 10 (−8) rs1801274: *P* = 1.6 × 10 (−6)	(141)
5	(142)	KD	*IGHV3-66* variant	IG heavy chain variable gene	rs4774175; OR = 1.20, *P* = 6.0 × 10 (−9)	(142)
6	(143)	KD	HLA-DRB1	Regulation of immune system	Development of CAL in KD	(143)
7	(144)	Severe COVID-19	HLA-B*46:01	Regulation of immune system	Computer simulation study predicted vulnerability to COVID-19	(144)
8	(144)	Severe COVID-19	HLA-B*15:03	Regulation of immune system	Allele allows preferential presentation of highly conserved domains	(144)

*CAL, coronary artery lesions; HLA, human leukocyte antigen; IGHV, immunoglobulin heavy variable; XIAP, X-linked inhibitor of apoptosis; CYBB, cytochrome b-245 beta.*

## Host Pathogen Interactions in COVID-19, Kawasaki Disease and Multisystem Inflammatory Syndrome in Children

### Pattern Recognition Receptors and Type I Interferons Response

Innate immune system confers protection against viral infections, including SARS-CoV, MERS-CoV and SARS-CoV-2 by acting as first line of defense (42, 43). Components of the CoVs trigger host innate immunity. Viral pathogen-associated molecular patterns (PAMPs) and damage associated molecular patterns (DAMPs) are recognized by endosomal as well as cytosolic pattern recognition receptors (PRRs). The viral spike-S protein, single-stranded and double-stranded RNA (ssRNA and dsRNA respectively) act as ligands for PRRs such as Toll Like Receptors (TLRs)-2, 3, 7/8, retinoic acid-inducible gene-I-like receptors (RIG1) and melanoma differentiation associated protein (MDA-5). These PRRs sense viral nucleic acid and activate downstream transcription factors including nuclear factor-kappa B (NF-κB), interferon regulatory factors (IRFs)-IRF3 and IRF7. These downstream transcription factors cause production of proinflammatory cytokines and induction of anti-viral type-I interferons (IFN). IRF3/7 homodimerize and translocate to nucleus and induce synthesis of type I IFNs (IFNα and IFNβ). Subsequently, IFNs bind to IFN α/β receptor (IFNAR) activating Jak/Stat pathway and trigger production of antiviral interferon-stimulated genes (ISGs). This mechanism may protect host cells from exacerbated viral replication and cellular damage (44).

TLR adaptor myeloid differentiation primary response 88 (MyD88) signaling activates nuclear factor kappa light-chain-enhancer of activated B cell (NF-kB) resulting in synthesis of pro-inflammatory cytokines. These cytokines further prime NOD-, LRR- and pyrin domain-containing protein 3 (NLRP3) inflammasome leading to production of pro inflammatory cytokine-IL-1β facilitating pyroptosis (45). These observations are in sync with a recent transcriptome profiling of respiratory epithelial cell lines infected with SARS-CoV-2. The study demonstrated a robust pro-inflammatory cytokine response and low IFN levels (46). ORF6, ORF9b, and ORF3b-encoded proteins in SARS-CoV-2, have been demonstrated to decrease antiviral type I IFN (IFN-I) production and signaling (21, 47, 48). Also, SARS-CoV-2 non-structural proteins (nsp) -3 and -6 were shown to suppress IFN activation through the IRF3 pathway (49). This initial postponement of IFN-I response is followed by unrestricted viral reproduction and dissemination in the infected host thereby resulting in a subsequent surge in IFN-I, which aggravates hyperinflammation and may predispose to a severe clinical illness (49).

Recent COVID-19 and complete KD transcriptome dataset analysis has reported downregulated TLR7, IRF3 and stimulator of interferon genes (STING) expression in KD patients (50, 51). SARS-CoV-2 viral infection mediated RAS activation, *via* Ang-II and Angiotensin II type 1 receptor (AT1R), also triggers the TLR4/MyD88/NFκB pathway resulting in elevated pro-inflammatory cytokines including TNF-α, IL-1β, IL-6 and IL-8 (22).

The cytokine profiling in MIS-C revealed down-regulation of tumor necrosis factor like weak inducer of apoptosis (TWEAK) (52), a negative regulator of IFN-γ. This indicates attenuation of IFN response and transition of innate to adaptive immunity (53). Lag in IFN response may result in delayed clearance of viral load and elevated inflammatory state (54) which may result in production of autoantibodies and alterations in self-tolerance. However, delay of 4–6 weeks in the development of MIS-C post-infection by SARS-CoV-2 indicates immune dysregulation may be the primary cause of MIS-C in predisposed children.

Clinically MIS-C overlaps with features observed in KD, TSS and secondary hemophagocytic lymphohistiocytosis (sHLH) (55). Similar to MIS-C, sHLH is characterized by immunological dysregulation, which includes immune hyperactivation, increased cytokine production, and severe systemic inflammation. Current HLH diagnostic criteria requires presence of at least five conditions including fever, splenomegaly, cytopenias, hypertriglyceridemia and/or hypofibrinogenemia, hemophagocytosis in bone marrow or spleen or lymph nodes, reduced or absent NK cell activity, elevated ferritin and soluble CD25 (56). It is also critical to investigate the infectious trigger in case of sHLH. Elevated IFN-γ, and IL-10 are important biomarkers in the diagnosis of HLH (57). However various independent studies indicate that primary (genetic cause) and secondary (infectious, malignant or autoimmune mediated trigger) HLH can be differentiated based on the levels of IFN-γ (56, 58, 59).

In a recent study, Esteve-Sole et al. compared IFN-γ levels between healthy controls, KD, MIS-C, and MAS. The cytokine profile of MIS-C and KD overlapped with elevated IFN response markers (IFN-γ, IL-18 and IP-10) and monocyte activation markers (MCP-1, IL-1α and IL-1RA). However, a subset of MIS-C cases differentiated to form a subgroup with markedly elevated IFN-γ with incipient indication to MAS (60). As per previous studies, the MIS-C subgroup had elevated IFN-γ levels which indicate the involvement of an IFN-regulated mechanism.

Low erythrocyte sedimentation rate (ESR) and splenomegaly usually seen in sHLH, are not observed in MIS-C (61, 62). The cytokine profile of patients with COVID-19 and sHLH was similar with elevated serum levels of IL1, IL2, IL4, IL6, IL7, IL8, IL10, IL-18, TNFα, GCSF, IP-10, MCP1, MIP1α, CXCL9, CXCL-10, IFN-γ (63–66). A comparison of immunological perturbations seen in SARS, MERS, COVID-19, KD and MIS-C is tabulated in Table 4.

**TABLE 4:** Immune mechanisms operative in SARS-MERS-COVID-19 infections and KD/MIS-C.

Immune component	SARS	MERS	COVID-19	KD	MIS-C
Entry into host cell	ACE-2 Co-receptors: DC-SIGN (CD209) L-SIGN (CD209L) (145)	CD26/Dipeptidyl peptidase-4 (DPP-4)	High affinity binding of ACE-2 (25). Alternate receptor: CD147-SP	Etiological agent not discovered yet	High affinity binding of ACE-2 (25)
Innate Immunity					
Inflammasome	NLRP3 inflammasome activation by SARS-CoV 3a protein (146)	NLRP3 inflammasome triggered by C5aR1	NLRP3 inflammasome IL-1β and IL-6 activation (147)Increased type 1 IFN STING/TBK1 (TANK-binding kinase 1)/IRF pathway	Dysregulated NLRP3 inflammasome (148) Downregulated STING expression	Upregulation of NLRP3 and Il-β signaling (14)
Monocytes	Poorly infect monocytes/ macrophages (149)	Poor replication in monocytes/ macrophages but significant anti-viral immune response (149)	Reduced monocyte subsets, lowered expression of HLA-DR, and elevated CD163 (150).	Elevated CD14^+^CD16^+^ monocytes in acute KD (151, 152)	Reduced CD14, elevated CD64 (FcRγ1) expression in non-classical CD16^+^ monocytes (85)
IFN-γ	Delayed and diminished levels of IFN-I.	Delayed and diminished levels of IFN-I.	Reduced IFN- γ in COVID-19 generates cytokine storms	Differential response as per host IFN-I polymorphism (153)	Dysregulated IFN-γ (154)
Complement/coagulation	C3 factor CR1 in erythrocytes	C5a in the serum C5b-9 in the lung tissues	Thrombotic microangiopathy; increase in C5b-9 levels (99)	Classical pathway: C3 and B activation	sC5b-9 leading to microangiopathy
Adaptive Immunity					
B-Cells	Antibody dependent enhancement	Attenuated B cell response	Antibody dependent enhancement	IgA^+^ peripheral B cells from acute KD (155) Elevated “activated” CD86^+^ B cells (156)	Reduced total, effector and class switched memory B cells. Auto-antibodies including endoglins, exclusively to MIS-C: MAP2K2 and casein kinase family.
T Cell	Lymphopenia and suppressed T-cell activation; long-lived memory CD4^+^ and CD8^+^ T-cell responses—Polyfunctional CD4^+^ and CD8^+^ T cell responses.	Lymphopenia; CD8^+^ T-cell response specific to MERS in severe disease; long-lived memory T-cell responses.	Lymphopenenia; reduction in functional diversity of T cells; higher exhaustion, reduced multi-functional CD4^+^ T cells and higher CD8 ^+^ T cell exhaustion	Increased CD69^+^ natural killer and γδ T-cells (155) Decreased follicular helper T cells in children having COVID -19	Decreased naive CD4^+^ T cells (CD4^+^CD45RA^+^) and elevated memory T cells (CD4^+^CD45RO^+^) Both CM and EM CD4^+^ T cells are noted in MIS-C but not in KD (157). Higher levels of senescent T cells (CD57^+^) were noted in MIS-C in comparison to pediatric mild COVID-19 cases, adults with COVID-19 and KD patients.
Cytokine Storm	Th17 mediated (158)	Th17 mediated (158)	Th17 mediated (158)	Elevated levels of Th17 mediated IL-17A in KD (160) compared to MIS-C patients	Consistent myeloid activation
Cytokines and chemokines	IFN TGF, IL1, IL6, IL8, IL-12, CCL2, CXCL3, CXCL5 CXCL9, CXCL10, MCP1	IFN TGF, IL1, IL6, IL8. CCL2, CCL3, CCL5, CXCL10	IL1, IL6, MCP1, IL-2, IL8, IL-7, IL-17, G-CSF, GM-CSF, MIP 1α	IL-6, IL-8, IL-1 and IL-17A levels	IFN-γ, IL-18, IL-1β, IL-8, IL-6, IL-10, IL17 and TNF

*DC-SIGN, dendritic cell-specific intercellular adhesion molecule-3-grabbing non-integrin.*

## Role of Inflammatory Cells in COVID-19, Kawasaki Disease and Multisystem Inflammatory Syndrome in Children

### Neutrophils, Monocytes and Antigen Presenting Cells

In a recent multi-omic study, several immunological perturbations were documented in fatal SARS-CoV-2 infections. Peripheral neutrophils have upregulated genes which are involved in the synthesis of pro-inflammatory cytokines, enhance phagocytosis and degranulation (67). There is induction of interferon-stimulated genes, leukocyte recruitment and cytokine induction *via* the Ca^+^ binding proteins, S100A8/A9.

### NETosis

Neutrophil extracellular traps (NETs) were also documented in severe COVID-19 infections (68). NETs are a combination of extracellular DNA, oxidase enzymes and microbicides released by neutrophils in order to contain the infection. The acetylation neutralizes positive charge on histones and allows the DNA to decondense which is crucial for NET formation. The Histone deacetylase2 (HDAC2) suppresses de-condensation and thus impairs NETs. The SARS-CoV-2 non-structural proteins, especially Nsp5, impact the formation of neutrophil extracellular traps (NETs). The viral protein, Nsp5 attenuates the HDAC2 and promotes NETs (69, 70). Along with S100A8/A9, peptidyl arginine deiminase-4 (PAD4) also facilitates NETosis *via* PAD4-mediated histone citrullination (67, 71). NET specific markers including myeloperoxidase-DNA (MPO-DNA), and citrullinated histone H3 (Cit-H3) have been found to be elevated in severe COVID-19 (68). NET formation has also been linked to vascular damage and organ dysfunction in COVID-19 (72). NETs are predictors of poor outcome in the patients with COVID-19 ARDS (51) and contribute toward increased mortality (68). Higher numbers of NETs have also been observed in acute KD compared to the convalescent KD indicating endothelial damage associated with this disease (73). Netosis may also be associated with enhanced vascular injury (74) and could be one of the pathogenic mechanisms of KD vasculitis. Considering the overlapping clinical and molecular features between KD and MIS-C, it was also hypothesized that the excessive NET formation may contribute to the severe cardiac manifestations in MIS-C (75). Seery et al. reported significantly elevated NETs in the MIS-C group compared to mild and moderate pediatric cases with COVID-19 (76). Hence, NET formation may act as critical indicator predicting vascular damage in MIS-C.

The elevated neutrophils also produces chemoattractants including CXCL2 and CXCL8 in bronchoalveolar lavage fluid (BALF) and PBMCs which signifies the role of neutrophil recruitment in severe COVID-19 (77, 78). Flow cytometry based study on monocytes from patients with severe COVID-19 demonstrated elevated levels of IL-6, IL-1, and TNF-α, indicating an inflammatory monocyte phenotype.

### Monocytes/Macrophages

In severe SARS-CoV-2 infections, elevated numbers of monocyte-derived macrophages were observed in BALF comprising 80% of all infiltrated cells. Macrophage infiltrates have been previously observed in the post-mortem lungs from SARS-CoV affected patients. Due to similar cytokine profiles, it was hypothesized that mononuclear phagocytes may contribute to the hyperinflammation associated with COVID-19 (65). Histopathological assessment in autopsied KD cases reveals elevated monocyte/macrophage numbers in coronary arterial lesions (79, 80). These macrophages produce inflammatory chemokines, such as granulocyte-macrophage colony-stimulating factor (GM-CSF) and IL-6, which result in increased recruitment of monocytes, macrophages and dendritic cells (81) and exacerbated immune response which may be associated with the cardiac manifestations. GM-CSF has also been observed to be critical in murine model of KD as it orchestrates cardiac inflammation (82). Thus, monocytes and neutrophils appear to play a central role in severe SARS-CoV-2 infections and KD. Monocytes induce cytokine storm and lung injury whereas neutrophils lead to enhanced chemotaxis, NETosis, and a hyper active immune response in severe SARS-CoV-2 infections.

**FIGURE 4:**
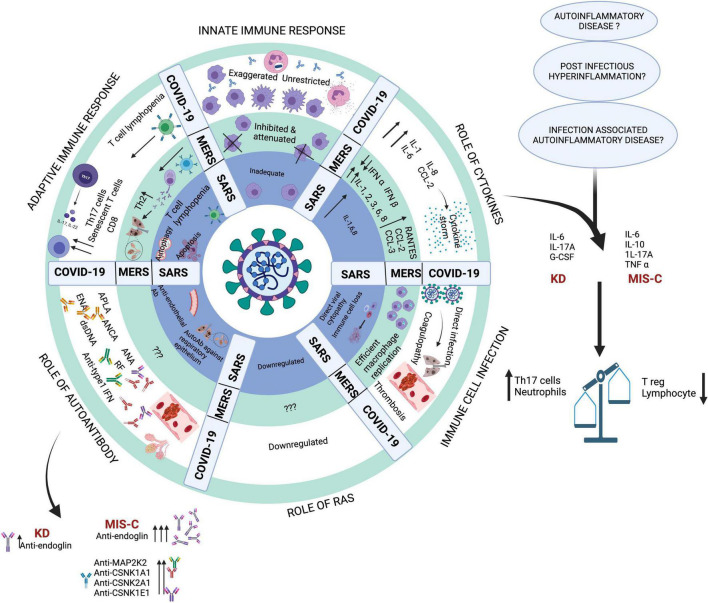
Key pathogenic mechanisms in SARS, MERS and COVID-19. Created with BioRender.com.

MIS-C presents as a global activation of neutrophils and non-classical monocytes evidenced by increased CD64 (FcγRI) and CD54 (ICAM1) expression pointing toward consistent myeloid activation (83). There is an increase in immature neutrophils (low CD10 and CD62L) in the peripheral blood of MIS-C patients (84). Despite global activation of the myeloid lineage, HLA-DR and CD86 were found to be reduced on monocytes and dendritic cells. This was accompanied by a concomitant decrease in natural killer (NK) cells (CD56 low), plasmacytoid dendritic cells, CD16^+^ monocytes, and γδ T lymphocytes (83, 85). Together, this may result in an impaired antigen presentation and a dysregulated immune response. Detailed immunopathogenesis mechanisms have been illustrated in Figure 4.

Dysregulated activation of neutrophils in addition to an enhanced expression of human neutrophil antigen 2 (HNA-2) encoded by the *CD177* gene, a specific activation marker of neutrophils, has also been noted in KD patients. CD177 enhances antimicrobial activity of neutrophils and an increased expression has also been associated with septic shock and inflammatory bowel disease. Abnormal methylation pattern of *CD177* gene and enhanced expression of CD177 in KD patients has been associated with intravenous immune globulin (IVIG) unresponsiveness (86). CD177 has also been associated with development of severe disease and mortality in COVID-19 (87).

### NK Cells

Severe SARS-CoV-2 infections present with activated NK cells but in patients progressing to fatal COVID-19, the proliferation of NK cells is impaired. There is decrease in the cytotoxic effector perforin as compared to patients with severe COVID-19 (88). This indicates functional alterations of NK cells in patients who succumb to the disease. Patients with SARS-CoV-2 infections have decreased IFNγ production in NK cells when compared to healthy controls (89). Witkowski et al. reported higher levels of anti-inflammatory molecule transforming growth factor-β1 (TGF−β1) indicating altered antiviral defense mechanism by NK cells (90). The untimely enhancement of TGF-β1 alters NK cell function and negatively impacts early control of viral clearance.

Initial reports on MIS-C described NK cell cytopenia (91). Beckmann et al. reported aberrant NK cells in MIS-C using a transcriptome based approach. This study reported partially shared molecular etiology with KD but not with other inflammatory conditions. In patients with MIS-C and KD, exhausted effector CD8^+^ T-cells and CD56*^dim^*CD57^+^ natural killer (NK) cells were reported (92). Another single-cell RNA sequencing-based study also reported enhanced expression of cytotoxicity genes in NK and CD8^+^ T cells (93). The elevated levels of cytolytic enzymes, such as perforin, granzyme A, and granzyme H may be responsible for tissue damage seen in MIS-C (93). These studies suggest sustained hyper inflammation may be due to depletion of NK cells leading to CD8^+^ T-cell exhaustion. In a murine model, NK cells eliminated viral activated CD8^+^ T-cells *via* Natural Cytotoxicity Receptor 1 (NCR1). This study indicated a cross-talk between these two cell types (94). The CD8^+^ T-cells are crucial in resolving post-viral infection-associated pathology. Thus, in the event of NK cell depletion, increased senescent CD8^+^ T-cells may be responsible for T cell-associated immunopathology observed in MIS-C patients (95, 96).

## Role of Complement Components

In SARS-CoV-2 infection, complement activation appears to play a key role (97). This is evidenced by endothelial deposition of complement and elevated levels of serum C5a in patients with severe COVID-19 (97). Yu et al. demonstrated the role of alternative complement pathway activation primarily mediated by SARS-CoV-2 spike proteins. They also reported that inhibition of C5 blocks the accumulation of C5b-9, but not C3c (98). Dorio et al. found increased levels of soluble C5b-9 (sC5b-9) in cases with MIS-C as well as severe COVID-19, but not in patients with milder forms of the disease (99). Elevations of sC5b-9, an activation product of the terminal complement cascade, have been linked to microangiopathy in a number of studies (100, 101). Surrogate markers of microangiopathy (as evidenced by burr cells and schistocytes) are seen in almost all patients with MIS-C and a significant proportion of patients with severe COVID-19 (99). Compared to healthy control and KD group, MIS-C reported highly enriched proteins associated with complement pathways specifically the components of classical complement cascade C1qA, C1qB, and C1qC (102). Hence, IVIg treatment is recommended to inhibit the complement deposition in MIS-C. Serum complement C3 is known to be elevated in KD (103). Role of C5a and C5b has also been implicated in the membrane damage associated in KD (104). The detailed comparison has been provided in Table 4.

## Role of T Cells in COVID-19, Kawasaki Disease and Multisystem Inflammatory Syndrome in Children

Patients with COVID-19 have shown an unbalanced T cell homeostasis with reduction of multi-function CD4^+^T cells and higher degree of CD8^+^T cell exhaustion, leading to reduction in T cell subsets and an ineffective cellular immune response (105). In severe SARS-CoV-2 infections, elevated numbers of CD8^+^ and Th17 cells were noted in peripheral blood and lung biopsy samples. This can explain lung injury seen in patients with severe COVID-19 (106). It appears that a strong Th17 mediated cytokine storm drives lung injury in COVID-19.

Patients with MIS-C and KD also present with decreased T cell counts and naïve CD4^+^ T cells (CD4^+^CD45RA^+^) but increased memory T cells (CD4^+^CD45RO^+^). A subset of memory T cells, viz. Central memory (CM) and effector memory (EM) CD4^+^ T cells were identified in MIS-C and pediatric SARS-CoV-2 cases in higher proportion when compared to KD (52). However, CD8 + effector memory cells were reduced in MIS-C compared to adult COVID-19 (107). Elevated CD4 + T cell proliferation was reported in MIS-C compared to the healthy subjects (93). A transcriptome based study revealed decrease in the CD8 + T cells and down-regulation of exhausted T (Tex) cells in the cases with MIS-C (92). It is crucial to emphasize the role of CD8 + T cells and NK cells which co-regulate one another to elicit cytolytic outcomes. A contrasting feature of MIS-C from COVID-19 is the T cells expressing fractalkine receptor (CX_3_CR1 + CD8 + T cells) which homes the cells to endothelium. This may also explain the endothelium abnormalities observed in MIS-C. Moreover, expression of these cells reduces during the improvement in clinical course (108). Increased number of senescent T cells (CD57^+^) had been noted in MIS-C, KD and in adults with mild COVID-19 infection (109). In contrast to senescent T cells, decreased numbers of follicular helper T cells were noted in children with SARS-CoV-2 infection and also in children who developed MIS-C.

A recent study on patients with MIS-C has shown polyclonal V21.3 T cell expansion directed toward SARS-CoV-2 antigenic peptides. This is unlike the lymphocyte phenotype seen in KD, TSS and COVID-19 patients. Alterations in T cell repertoire resolved after recovery from MIS-C (110). In another study, Porritt et al. have hypothesized the role of bacterial superantigens in development of MIS-C and observed T cell expansion of TCRβ variable gene 11-2 (TRBV11-2) (111). GM-CSF is also believed to have a role in pathogenesis of MIS-C. Previous studies had associated MIS-C with autoimmunity (85, 112). The study by Beckman et al., interrogated autoimmune signatures from transcriptome study databases but MIS-C was not associated with alternations in autoimmune pathways (92). This hints that novel autoimmune mechanisms may govern the predisposition and disease course for MIS-C. It has been noted that in KD patients, GM-CSF-activated myeloid cells release pro-inflammatory cytokines which may be associated with disease pathology. This also increases dendritic cell priming of T cells during antigen-specific immune responses (113).

## Multisystem Inflammatory Syndrome in Adults

Multisystem inflammatory syndrome in adults (MIS-A) was also reported, although only few cases were seen (114). The common pathogenic hallmarks associated with MIS-C/A could be macrophage hyperactivation, autoantibodies and antigen-antibody complexes. Moreover as emphasized earlier, MIS-C/A could be triggered in genetically predisposed individuals. Ronit et al. reported impaired Type I IFN (IFNα and IFNβ) and type III IFN (IFNλ) response in comparison to healthy controls, however, the response did not differ from severe COVID-19 cases admitted in ICU. Whole exome sequencing of MIS-A cases revealed variants in the genes associated with autophagy, Kawasaki disease, immune responses and viral restriction factors (115). In contrast to the cases with severe COVID-19 and MIS-C, IL-6 and IL-1β levels were normal in MIS-A cases (115, 116). Kawasaki disease in adolescent group is incredibly rare, however, KD-like features have been observed in MIS-A group.

## B Cells and Antibody Responses in COVID-19, Kawasaki Disease and Multisystem Inflammatory Syndrome in Children

In most instances MIS-C results from an exaggerated inflammatory response that occurs several weeks after apparently asymptomatic or mild SARS-CoV-2 infection. Patients have neutralizing antibodies to SARS-CoV-2. In a recent study, MIS-C patients were immuno-profiled and B cell cytopenia was reported. Lower levels of B cell subsets, viz. effector B cells (plasma cells) and class switched memory B cells, were seen (117). SARS-CoV-2 infected infants had a lower number of follicular T helper (Tfh) cells which play crucial role in B cell maturation. However, alterations of Tfh cell frequencies were not observed in patients with KD (52). Woodruff et al. observed extrafollicular B-cell responses characterized by loss of germinal centers, expanded B cells and plasmablasts, and altered Bcl-6 expression in severe cases with COVID-19 (118). In acute KD, proportion of CD138^+^ and IgG^+^ antibody secreting plasmablast cells were increased while frequency of memory B cells was decreased (119, 120). T-box transcription factors T bet and eomesodermin expression was higher on plasmablasts in MIS-C compared to pediatric COVID-19. However, frequencies of plasmablasts were observed to be similar in both groups. This indicates an altered differentiation state of plasmablasts in MIS-C compared to pediatric COVID-19. MIS-C has also been associated reduced IgA responses and persistent IgG production leading to activation of monocytes (121).

Onset of MIS-C coincides with peak antibody production. A recent study reported presence of anti-spike (S) IgG, IgM, and IgA antibodies, as well as anti-nucleocapsid (N) IgG antibodies in an adult COVID-19 cohort (122). Children with COVID-19 and MIS-C, on the other hand, displayed a restricted repertoire of anti-SARS-CoV-2 specific antibodies, generating predominantly IgG antibodies specific for the S protein but not the N protein. Moreover, lowered neutralizing activity was observed in both MIS-C and non-MIS-C groups in comparison to the adult COVID-19 cohort. Reduced anti-N antibodies correlate with the milder disease course in pediatric cohort, unlike adult cases with COVID-19 where higher levels of anti-N antibodies have been reported. Higher anti-N antibodies also correspond to higher viral cell lysis (122).

Autoimmunity is also implicated in pathogenesis of SARS. Autoantibodies against respiratory epithelial cells and endothelial cells are implicated in both cytotoxic injury of respiratory epithelium as well as systemic vasculitic pathology observed in SARS infection (123). Exposure of autoantigens due to organ injury or development of cross-reacting antibodies to SARS-CoV epitopes may be responsible for production of autoantibodies (124).

## Autoantibodies in COVID-19, Kawasaki Disease and Multisystem Inflammatory Syndrome in Children

In severe COVID-19 there is multi organ involvement that may be accompanied by thrombotic lesions in heart and lungs. Several auto-antibodies have been associated with the thrombosis encountered in patients with COVID-19. These include anti-cardiolipin (a-CL), lupus anti-coagulant, and anti- β2GPI, anti-neutrophil cytoplasmic antibodies, anti-nuclear antigen, anti-rheumatoid factor, anti-cyclic peptide containing citrulline peptide, antibodies to extractable nuclear antigen and anti-double stranded DNA (125). Accumulating evidence of pre-existing autoantibodies against type I IFNs was first provided by Bastard et al. and a sharp increase in neutralizing type I IFNs autoantibodies was seen in the patients above 70 years (126, 127). This could be another factor favoring the hypothesis of genetic predisposition to severe COVID-19 and may explain the higher mortality in the geriatric population.

In a recent study, the levels of autoantibodies in COVID-19 cases were observed to be as high as 50% compared to 15% of healthy control group (128). This sporadic loss of self-tolerance may explain the mortality associated with severe COVID-19. Auto-antibodies targeting connective tissues and cytokines may be associated with the severity of COVID-19. The longitudinal production of IgG and auto-antibodies might be correlated with the viral genome and non-structural proteins of SARS-CoV-2 (128). In another study, Bastard et al. have reported anti-IFN auto-antibodies in 101 of 987 cases with severe SARS-CoV-2 infection. These included anti-IFN-ω in 18/101, anti-IFN-α in 36/101 and both anti-IFN-ω and anti-IFN-α in 56/101 cases. Patients with mild and asymptomatic infection, however, did not have measurable autoantibodies (125). Similarly, Goncalves et al. reported positive IFN-I auto-Abs in 18% severe COVID-19 cases, IFN subtypes IFN-ω and no measurable IFN-I auto-Abs in mild cases (129). In a French study, auto-Abs were observed in 20% severe COVID-19 cases (130). Studies have also reported the anti-cytokine antibody (ACA) blocking activity in severe COVID-19 (125, 131, 132). Moreover, presence of anti-IFN antibodies may significantly impair the immune response itself and may lead to a broader immune dysregulation. This was previously seen in inborn errors of immunity such as diseases of immune dysregulation presenting with monogenic and polygenic defects (*RAG1/2*, *AIRE*, auto-antibodies against *IL-17*, *IL-22*, *IFN-*γ, and GM-CSF) (133). ACAs have also been observed in SLE patients, thus they play a critical role in maintaining homeostasis of immune response. Similarly, ACAs play an important role in COVID-19 which may alter the immune response in these patients (128). On the other hand, the activation of pro-inflammatory markers and auto-antibodies may dampen T-cell responses resulting in altered self-tolerance.

Elevated levels of autoantibodies targeting endoglins were reported in several cases with MIS-C using proteomic arrays (52). Endoglin glycoprotein is expressed by endothelial cells and is critical in maintaining structural integrity of arteries. Autoantibodies against endoglin have also been reported in a small proportion of cases with KD (52). However, autoantibodies against *MAP2K2* and casein kinase family (viz. *CSNK1A1, CSNK2A1, CSNK1E1*) were only found in the cases with MIS-C. Gruber et al. have also reported the role of autoantibodies targeting endothelial, gastrointestinal, and immune-cell (85).

Mechanism of autoantibody clearance in MIS-C has not been explored thoroughly. As described earlier, low levels of TFH cells are reported in acute phase of MIS-C. Reduced TFH cells are associated with generation of low affinity antibodies. These, low affinity antibodies are cleared rapidly than the high affinity antibodies.

### Clinical Management of Multisystem Inflammatory Syndrome in Children

As described above, immunopathogenic alterations in MIS-C have been reported that have prompted recommendations for diverse treatment strategies. Patients with MIS-C have an enhanced propensity to vascular damage and hyperinflammation, therefore, aggressive management of cardiac manifestations and immunomodulation is recommended (134). MIS-C patients with CAA require frequent echocardiograms. Cardiac MRI is indicated in cases with left ventricular (LV) dysfunction and in patients with suspicion of distal CAAs Cardiac CT is recommended. Immunomodulatory treatments are required in MIS-C cases with life-threatening complications. IVIg therapy in MIS-C accelerates auto-antibody clearance, inhibition of complement deposition, and increase in T-regulatory cells (135). The use of biologics has been recommended for cases refractory to treatment with IVIG and corticosteroids (134, 136, 137).

## Conclusion

In this manuscript, we have reviewed the putative immunopathogenic mechanisms operational in SARS, MERS, COVID-19, MIS-C and KD. SARS-CoV-2 is characterized by a modified spike (S1) polypeptide that has a higher binding affinity to host NRP1 receptors. This is responsible for the increased infectivity and tissue tropism seen with SARS-CoV-2 infections. Patients with COVID-19 appear to have delayed IFN responses that results in upregulation of the cytokines IL-6, IL-7 and TNF-α. This aggravates inflammation and may exacerbate clinical illness.

MIS-C, previously considered a KD-like disease, differs by age of presentation, ethnicity and immune response. Moreover, MIS-C is characterized by a differential cytokine production (IL6 and IL10), altered differentiation state of plasmablasts (T-bet, eomesodermin), and auto-antibodies to MAP2K2, CSNK1A1, CSNK2A1 and CSNK1E1. Host genetic factors also appear to play a crucial role in predisposition to MIS-C. Variations in *SOCS1, XIAP* and *CYBB* genes have been reported to be associated with pathogenesis of this disorder. These variations are different than the loci previously associated with development of KD.

There is concern about the long term cardiovascular sequalae of MIS-C as children present with acute myocardial injury/myocarditis and may develop coronary artery aneurysms. Differences in the immune response to CoVs between adults and children could partly explain the difference in the disease severity observed across age groups. Although the pathogenesis of MIS-C is not completely defined, it is reasonable that age related peculiarities in the immune response to CoVs could be involved to explain its prevalence in the pediatric population. Thus, MIS-C and KD are a spectrum of hyperinflammatory disorders arising as a sequel to known (MIS-C) and unknown (in KD) infections. Deeper understanding of these disorders is required to resolve the relationship between MIS-C and KD.

## Author Contributions

SS: conceptualization of the review, substantial contribution in drafting, and finalizing the draft. AR: substantial contribution in drafting and revising the draft. MD, RT, and PM: substantial contribution in drafting and revising the draft at various stages. PB, SL, JS, KS, and SM: substantial contribution in drafting the manuscript.

## Conflict of Interest

The authors declare that the research was conducted in the absence of any commercial or financial relationships that could be construed as a potential conflict of interest.

## Publisher’s Note

All claims expressed in this article are solely those of the authors and do not necessarily represent those of their affiliated organizations, or those of the publisher, the editors and the reviewers. Any product that may be evaluated in this article, or claim that may be made by its manufacturer, is not guaranteed or endorsed by the publisher.
